# CDK11 inhibition induces cytoplasmic p21^WAF1^ splice variant by p53 stabilisation and SF3B1 inactivation

**DOI:** 10.1002/1878-0261.70143

**Published:** 2025-10-17

**Authors:** Radovan Krejcir, Lukasz Arcimowicz, Lucia Martinkova, Vaclav Hrabal, Filip Zavadil Kokas, Tomas Henek, Martina Kucerikova, Ondrej Bonczek, Pavlina Zatloukalova, Lenka Hernychova, Philip J. Coates, Borivoj Vojtesek, David P. Lane

**Affiliations:** ^1^ RECAMO Masaryk Memorial Cancer Institute Brno Czech Republic; ^2^ National Centre for Biomolecular Research, Faculty of Science Masaryk University Brno Czech Republic; ^3^ Laboratory of Growth Regulators, Institute of Experimental Botany The Czech Academy of Sciences Olomouc Czech Republic; ^4^ Department of Microbiology, Tumor and Cell Biology Karolinska Institute Solna Sweden

**Keywords:** cyclin‐dependent kinase 11, MDM2, p21^L^, p21^WAF1^, p53, SF3B1

## Abstract

CDK11 is a cyclin‐dependent kinase with a role in transcription and RNA splicing and represents a potential target for cancer treatment. We show that blocking CDK11 activity with the OTS964 inhibitor causes p53 stabilisation through MDM2 downregulation. Under these conditions, p53 activates the expression of its downstream effector *CDKN1A* (p21^WAF1^), produced in two isoforms, the canonical p21^C^ and the recently described p21^L^. We compared the ability of both isoforms to block proliferation and showed that p21^L^ partially lost its inhibitory potential, likely due to the missing cyclin‐binding Cy2 and PCNA‐interacting motifs and its cytoplasmic localisation. We identified the epitopes of four p21^WAF1^ antibodies using phage display to determine isoform specificity. Moreover, we show that the trigger for p21^L^ induction is inhibition of the spliceosomal protein SF3B1. CDK11 activates SF3B1 by phosphorylation, and inhibition of either SF3B1 or CDK11 induces p21^L^. We discovered an isoform similar to human p21^L^ in murine cells, suggesting evolutionary conservation of *CDKN1A* alternative splicing. Our results uncover an unknown link between RNA splicing and proliferation control involving a novel isoform of a key cell cycle inhibitor.

## Graphical abstract

CDK11 inhibition stabilises the tumour suppressor p53 and triggers the production of an alternative p21^WAF1^ splice variant p21^L^, through the inactivation of the spliceosomal protein SF3B1. Unlike the canonical p21^WAF1^ protein, p21^L^ is localised in the cytoplasm and has reduced cell cycle‐blocking activity. These findings reveal a new mechanism linking proliferation control and RNA splicing.

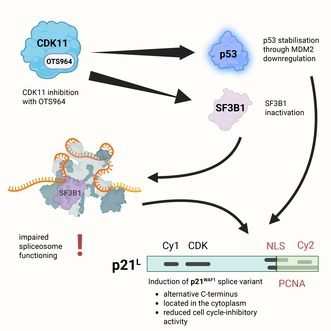

**Abbreviations**
AA: amino acid
CDK: cyclin‐dependent kinase
FA: formic acid
FACS: fluorescence‐activated cell sorting
GFP: green fluorescent protein
HCD: higher‐energy collisional dissociation
HDR: homology‐directed repair
LDS: lithium dodecyl sulphate
MDS: myelodysplastic syndrome
NLS: nuclear‐localisation signal
PARP: poly (ADP‐ribose) polymerase
PBS: phosphate‐buffered saline
SDS/PAGE: sodium dodecyl sulphate‐polyacrylamide gel electrophoresis
WT: wild‐type

## 1 Introduction

Small‐molecule inhibitors of cyclin‐dependent kinases (CDKs) have been observed to induce p53 levels in cells [1, 2]. Individual CDK family members regulate not only distinct steps in the cell cycle but also DNA transcription, and disruption of CDK functions represents a major stress for cells and compromises their genome integrity. Therefore, it is logical that the p53 protein, the so‐called guardian of the genome, is activated in response to CDK inhibition [3]. Moreover, concerning the role that CDKs play in transcription, it is noteworthy that p53 also earned the title of a sensor of transcriptional integrity [4]. The p53 protein has been the subject of intense research for many decades and numerous functions have been identified in various cellular contexts. However, at the centre of p53 activities remains the maintenance of genome integrity by sensing transcriptional inhibition and DNA damage, subsequently activating DNA repair processes and eliminating mutant cells to prevent cancer development [5, 6, 7].

The *TP53* gene is the most frequently mutated gene in cancer, and the functions of mutant p53 protein are compromised. However, about half of human cancers retain wild‐type (WT) protein and show p53‐dependent patterns of transcription indicating retained function [8, 9]. In this study, we focused on the wild‐type p53 protein and its interactions. In unstressed cells, p53 levels are low due to the activity of its main negative regulator MDM2, but in stress conditions, p53 is quickly stabilised. Small‐molecule inhibitors are available that disrupt the MDM2‐p53 binding, directly stabilising p53. Interestingly, these p53‐activating drugs show synergistic toxic effects against tumour cells in combination with CDK inhibitors [10, 11, 12, 13]. This phenomenon has not been explained completely, although it is assumed that simple p53 stabilisation is not sufficient for inducing a strong pro‐apoptotic response, and other events involving members of relevant signalling pathways are required [11].

CDK11 is a less extensively studied CDK that belongs to the subfamily of transcription‐regulating CDKs. There are two highly similar genes, *CDK11A* and *CDK11B*, in the human genome that encode an almost identical full‐length 110 kDa protein, p110, and several shorter isoforms with specific functions [14]. L‐type cyclins have been discovered to be the primary cyclins that bind CDK11 [15]. The study of CDK11 functions has been facilitated by the discovery of the CDK11‐specific inhibitor OTS964 in 2019 [16]. OTS964 showed remarkable selectivity, which is rare among the CDK inhibitors that usually target more than one CDK family member [16, 17]. Importantly, OTS964 showed high levels of activity in mouse xenograft models of human cancer [18]. One of the key functions of CDK11, likely relevant for OTS964's anticancer properties, is the complex regulation of gene expression by phosphorylation of the regulatory C‐terminal domain (CTD) of RNA polymerase II [17, 19]. Moreover, CDK11 is a fundamental player in regulating RNA splicing because spliceosome activation requires CDK11‐catalysed phosphorylation of the SF3B1 spliceosomal protein [17, 20]. In this context, it is of interest that RNA splicing is considered a possible target of cancer treatment based on the fact that tumour cells tend to be more sensitive than normal cells to splicing inhibition [21, 22, 23]. Even though mutations of spliceosome components are relatively common across tumour types and lead to multiple types of errors (e.g. splice‐site shifting or intron retention), they do not cause general suppression of RNA splicing. Among the genes coding for spliceosomal proteins, *SF3B1* is the most commonly mutated in cancer [24, 25].

The present work investigated the relationship between CDK11 and p53, and the role of p53 stabilisation in the toxicity of OTS964. Our experiments showed alternative splicing of *CDKN1A* (p21^WAF1^, hereinafter referred to as p21), a major downstream effector of p53 [26, 27, 28]. p21 belongs to the CDK interacting protein/kinase inhibitory protein (CIP/KIP) family of CDK inhibitors and represents a key player in cell cycle regulation. The classical model was based on p21 disrupting the binding of cyclins to several CDKs including CDK1, CDK2, CDK4, and CDK6. However, more recent studies showed that p21‐CDK binding is not always inhibitory and its effect depends on the context as well as p21 levels [28].

p21 physically interacts with CDKs and cyclins via separate amino acid (AA) motifs. For cyclin binding, two Cy motifs were identified, with the N‐terminal Cy1 (AA 17–24) showing stronger binding than the C‐terminal Cy2 (AA 152–158) [29, 30]. The CDK interacting motif is localised to AA 53–58 [29, 31]. A third key interaction partner of p21 is PCNA, where p21 competes with other PCNA interactors and lowers the amount of available protein, thus inhibiting DNA replication and repair [27]. The PCNA‐interacting protein (PIP) box motif is localised at the C‐terminus of p21 (AA 140–164) and makes p21 a stronger interaction partner for PCNA than most proteins [32]. This PIP box motif overlaps with the nuclear‐localisation signal (NLS) of p21 (AA 140–156) [33]. Even though p21 predominantly accumulates in the nucleus, it can be localised in the cytoplasm, and specific phosphorylation events in the NLS or NLS‐adjacent regions cause nuclear‐to‐cytoplasmic relocation (e.g. T145) [28, 34].

Despite p21 acting as a powerful inhibitor of the cell cycle, multiple lines of evidence show that p21 can have tumour‐promoting effects under specific circumstances. Importantly, the tumour suppressive/oncogenic behaviour of p21 seems to correlate with its nuclear/cytoplasmic localisation, respectively [28, 35]. An alternative isoform of p21 was described recently in cells exposed to the splicing inhibitor E7107 that targets SF3B1 [36]. This new isoform, called p21^L^, is translated from mRNA with an additional retained intron and has an alternative C‐terminus starting from AA 149. Thus, the PIP box motif, NLS, and Cy2 are disrupted or missing in p21^L^, which as a consequence accumulates in the cytoplasm and loses the ability to arrest the cell cycle. Therefore, the induction of p21^L^ would be an unwanted effect of cancer treatment with splicing inhibitors like E7107. Moreover, this isoform would theoretically represent a way to escape the tumour‐suppressive effects of p21 for tumours with *SF3B1* mutations mimicking the effects of the E7107 inhibitor.

## 2 Materials and methods

### 2.1 Cell culture

Cell lines A375 (RRID: CVCL_0132), HCT‐116 (RRID: CVCL_0291), RPE1 (RRID: CVCL_4388), and NIH3T3 (RRID: CVCL_0594) were maintained in standard culture conditions (37 °C, humidified atmosphere, and 5% CO_2_) in high‐glucose Dulbecco's modified Eagle's medium with 1% sodium pyruvate (A375, RPE1, NIH3T3) or McCoy's 5A medium (HCT‐116 cell line), both purchased from Merck (Darmstadt, Germany). The media were supplemented with 10% fetal bovine serum (Thermo Fisher Scientific, Waltham, MA, USA) and a penicillin/streptomycin antibiotic mixture (Biosera, Cholet, France).

The cell lines are regularly tested in‐house for mycoplasma contamination using a PCR test and authenticated with an STR‐based method.

The following chemical inhibitors were used: OTS964 and nutlin‐3a (Selleck Chemicals, Houston, TX, USA), and pladienolide B and TG003 (MedChemExpress, Monmouth Junction, NJ, USA). All inhibitors were dissolved in DMSO. Cells were treated with the indicated concentrations of the inhibitors, and control samples were treated with DMSO at a concentration equal to that applied with the highest concentration of the inhibitor.

### 2.2 Preparation of modified cell lines

A375 p53‐KO cell line was a gift from Prof. Ted Hupp from the University of Edinburgh. The cell line was prepared as described in Ran et al. [37]. The preparation of HCT‐116 p53‐KO cell line (originally named HCT116 p53^−/−^) was described in Bunz et al. [38].

A375 cells with inducible expression of p21^C^ and p21^L^ were prepared by stable transfection with a tetracycline‐inducible expression vector containing p21 cDNAs (synthetic DNA purchased from Thermo Fisher Scientific). The expression vectors were based on the pcDNA3.1 backbone and were prepared using Gateway technology (Thermo Fisher Scientific). A two‐plasmid Tet‐On system was used, with p21 cDNAs under the control of the tetracycline‐responsive promoter element, and a helper plasmid encoding the tetracycline‐sensitive transcriptional activator rtTA‐VP16 (G72P). The PiggyBac transposase system was used to facilitate the incorporation of the expression cassette into the genome. Cells were transfected using Lipofectamine 3000 (Thermo Fisher Scientific) according to the manufacturer's instructions. Cells were selected with puromycin dihydrochloride at 5 μg·mL^−1^ (GoldBio, St Louis, MO, USA) for 5 days and then with blasticidin S hydrochloride at 5 μg·mL^−1^ (GoldBio) for 5 days. After that, cells were sorted using FACS Aria III (BD Biosciences, Franklin Lakes, NJ, USA) to select those with high p21 expression based on GFP signal after induction with doxycycline hyclate (GoldBio) (5 μg·mL^−1^, 24 h). The GFP‐p21 transcriptional fusion provided information about the expression of p21 without direct protein–protein fusion. Finally, clones were established from the pool of inducible cells.

For transient transfection of A375 cells with p21, p21^C^, and p21^L^ cDNAs were cloned in a plasmid expression vector using Gateway technology. The pcDNA3.1‐based vector contained p21^C^ or p21^L^ cDNA with GFP in transcriptional fusion under the control of the EF‐1α promoter. The cells were transfected using Lipofectamine 3000.

HCT‐116/*CDK11B*(G579S) cell line was prepared according to Lin et al. [16]. Single AA substitution G579S was introduced into the *CDK11B* gene of HCT‐116 cells using HDR‐mediated gene editing. The *CDK11B* gene was cleaved by CRISPR/Cas9 and a single‐stranded oligodeoxynucleotide (ssODN) with G579S substitution was provided as a template for HDR. LentiCRISPRv2 plasmid [39, 40] was used to induce the expression of Cas9 and *CDK11B*‐targeting sgRNA (guide sequence GGTGACTTCGGGCTGGCGC) in cells after transfection. Cells were transfected with lentiCRISPRv2 and ssODN using Lipofectamine 3000. After 24 h, the transfected cells were treated with 400 nm OTS964 for 7 days to select the resistant cells with *CDK11B*(G579S) substitution. Control cells transfected with lentiCRISPRv2 plasmid without the ssODN template were all dead after 7 days with OTS964.

**Table:** 

*CDK11B*(G579S) ssODN:	
CCGGGGCCCACTCACAGCCGCCCATCTGTCGTTGCAGGTGAGTGACTTTGGACTGGCACGCGAGTACGGATCCCCTCTGAAGGCCTACACCCCGGTCGT	

### 2.3 Immunoblotting

A detailed description of sodium dodecyl sulphate‐polyacrylamide gel electrophoresis (SDS/PAGE) and blotting on nitrocellulose membrane was published earlier [41]. Sample preparation was as follows: cells were seeded in cell culture dishes and left to adhere for 24 h. The following day, cells were subjected to experimental treatment (transfection, application of an inhibitor) and incubated for the required time. Cells were harvested using lithium dodecyl sulphate (LDS) lysis buffer (4% LDS, TRIS‐buffered). Protein concentration was measured with the DC Protein Assay (Bio‐Rad, Hercules, CA, USA), and the samples were completed with mPAGE 4× LDS Sample Buffer (Merck) and dithiothreitol to the final 100 mm concentration. Following SDS/PAGE and blotting, membranes were incubated with primary antibodies and horseradish peroxidase (HRP)‐conjugated secondary antibodies diluted in 5% non‐fat dried milk in phosphate‐buffered saline (PBS) with 0.1% Tween 20 (Merck), except for the P‐H2A.X S139 antibody that was diluted in 3% bovine serum albumin in PBS with 0.1% Tween 20. Chemiluminescence was developed using enhanced chemiluminescence (ECL) solution and visualised with the ChemiDoc System (Bio‐Rad).

The following antibodies were used for immunoblotting: p53 DO‐1, MDM2 4B2, MDMX 3.1, and p21 Ab‐118 were produced in‐house; others were purchased from indicated vendors: p21 (12D1), PARP1 (9542), P‐H2A.X S139 (Cell Signaling Technology, Danvers, MA, USA), H2A.X (3E11B2) (Proteintech, Rosemont, IL, USA), β‐actin MA1‐140 (Thermo Fisher Scientific), p21 F‐5 (Santa Cruz Biotechnology, Dallas, TX, USA), and p21 Ab‐1 (Merck).

### 2.4 MDM2 mRNA quantification with RT‐qPCR

Total RNA was extracted from control A375 cells and A375 cells treated with OTS964 (200 nm, 24 h) using the RNeasy kit (Qiagen, Venlo, the Netherlands). Reverse transcription was performed using SuperScript™ IV VILO™ Master Mix (Thermo Fisher Scientific) according to the manufacturer's instructions. PowerUp™ SYBR™ Green Master Mix (Thermo Fisher Scientific) and QuantStudio 5 qPCR cycler (Thermo Fisher Scientific) were used for quantitative PCR. β‐actin served as an endogenous control for data normalisation. The relative levels of *MDM2* mRNA were calculated using the 2^−ΔΔCT^ method, and the data represent means of four technical replicates. graphpad prism 8 (GraphPad Software, San Diego, CA, USA) was used for data analysis and graph preparation. The sequences of primers used in this experiment are listed below.

**Table:** 

actin‐F:	5′‐GCCGACAGGATGCAGAAGGAG‐3′
actin‐R:	5′‐CTAGAAGCATTTGCGGTGGAC‐3′
MDM2 (254–275)‐F:	5′‐TGTTTGGCGTGCCAAGCTTCTC‐3′
MDM2 (344–366)‐R:	5′‐CACAGATGTACCTGAGTCCGATG‐3′
MDM2 (711–732)‐F:	5′‐TCAGGATTCAGTTTCAGATCAG‐3′
MDM2 (893–914)‐R:	5′‐CATTTCCAATAGTCAGCTAAGG‐3′

### 2.5 Epitope mapping with phage display

Phage display and data analysis were performed as described elsewhere [42]. In brief, immobilised antibodies were incubated with Ph.D.™‐12 Phage Display Peptide Library (New England Biolabs, Ipswich, MA, USA) at a concentration of 2 × 10^10^ pfu per sample. Unbound phages were washed away before elution in 0.1 m glycine pH 3. A sequencing library was prepared from the eluted phage with three PCR steps, and the library was sequenced using Illumina Nextseq 550 (Illumina, San Diego, CA, USA). The data were independently analysed using two approaches: phal software [43] was used for template‐based analysis, and hammock software [44] for template‐free analysis. The results from Hammock were visualised with WebLogo [45].

### 2.6 Fluorescence microscopy

For subcellular localisation analysis of p21 isoforms, A375 cells with inducible p21^C^ or p21^L^ expression and HCT‐116 cells treated with OTS964 were examined by immunofluorescence microscopy. A375 cells were seeded on 8‐well slides with removable chambers (Ibidi, Gräfelfing, Germany) at a density of 7.5 × 10^3^ cells per well, and after 24 h, p21 expression was induced with doxycycline hyclate (5 μg·mL^−1^). Following 24 h of induction, cells were fixed and permeabilised with an ice‐cold methanol/acetone mixture (1 : 1, v/v) for 10 min at 4 °C. Non‐specific binding was blocked with 3% bovine serum albumin (BSA) in PBS for 1 h at room temperature. Cells were incubated with anti‐p21 12D1 primary antibody diluted 1 : 200 in 3% BSA overnight at 4 °C. After washing three times with PBS, cells were incubated with Alexa Fluor Plus 647‐conjugated goat anti‐rabbit IgG secondary antibody (Thermo Fisher Scientific) diluted 1 : 500 in 3% BSA for 2 h at room temperature in the dark. Slides were washed three times with PBS and mounted using ProLong Glass Antifade Mountant with NucBlue (Thermo Fisher Scientific) for nuclear counterstaining. Slides were cured for 24 h at room temperature before imaging.

HCT‐116 cells were seeded on 8‐well chamber slides at a density of 1.5 × 10^4^ cells per well, and after 24 h, cells were treated with OTS964 and incubated for 24 h. Cell fixation, permeabilisation, and immunofluorescence labelling were performed as described above for A375 cells.

Images were captured using a fluorescence microscope (Eclipse Ti‐E; Nikon, Tokyo, Japan) equipped with a 60× oil immersion objective. For each condition, at least 100 cells from two independent experiments were analysed. Representative images were processed using nis‐elements ar software (version 5.21.02), Nikon, Tokyo, Japan.

### 2.7 Resazurin viability assay

Viability measurements were performed in a 96‐well plate format. Cells were seeded at a density of 1 × 10^4^ (HCT‐116) or 7 × 10^3^ (A375) cells per well and after 24 h were treated with OTS964. After 72 h of incubation, the culture medium was replaced by fresh medium containing resazurin sodium salt (100 mg·L^−1^) (Merck) and cells were incubated for 3 h at 37 °C. Fluorescence of accumulated resazurin metabolite resorufin was determined on a microplate reader (Tecan Infinite M1000 Pro; Tecan Group Ltd., Männedorf, Switzerland) (λ_ex_ = 570 nm, λ_em_ = 600 nm). The data represent means of six technical replicates. graphpad prism 8 was used for data analysis and graph preparation.

### 2.8 Measuring cell confluency with the Incucyte S3 instrument

Transiently transfected A375 cells expressing p21 were seeded into 96‐well plates using FACS Aria III cell sorter at a density of 4 × 10^3^ cells per well and monitored on the Incucyte S3 (Sartorius AG, Göttingen, Germany) for 5 days. Every 3 h, four images per well were captured in phase contrast mode using a 10× objective. A375 clones with doxycycline‐inducible p21 expression were seeded at a density of 3 × 10^3^ cells per well and left to adhere for 8 h. Cells were treated with 5 μg·mL^−1^ doxycycline and monitored for 5 days. Four images per well were captured every 6 h in phase contrast mode using a 10× objective. Data were analysed with the Basic Analyzer in the Incucyte 2022B Rev2 software (Sartorius AG, Göttingen, Germany) to determine cell confluence, and the results represent means of three technical replicates.

### 2.9 Mass spectrometry proteomic analysis of p21 isoforms

Samples were separated with SDS/PAGE, the gel was stained with Coomassie brilliant blue, and bands corresponding to 15–35 kDa were cut from the gel.

#### 2.9.1 In‐gel digestion

Gel pieces were washed with deionised water, cut into smaller pieces to facilitate buffer exchange, and decoloured with a freshly prepared 200 mm solution of ammonium hydrogen carbonate (NH_4_HCO_3_, pH 7.8) in 40% (v/v) acetonitrile for 20 min at 30 °C and equilibrated in 50 mm NH_4_HCO_3_ (pH 7.8) in 5% (v/v) acetonitrile for 30 min at 30 °C. The supernatant was removed, and gel pieces were dehydrated with acetonitrile. The supernatant was removed, and the samples were reduced with 10 mm DTT for 1 h at 60 °C, followed by alkylation with 55 mm iodoacetamide in the dark for 45 min at room temperature. The supernatant was removed, and the gel pieces were washed three times with equilibration buffer and dehydrated with acetonitrile. Trypsin digestion was carried out at 37 °C overnight using sequencing‐grade trypsin (Promega, Madison, WI, USA). Digested peptides were extracted using acetonitrile, vacuum dried, and desalted using C‐18 micro spin columns (Harvard Apparatus, Holliston, MA, USA) according to the manufacturer's guidelines.

#### 2.9.2 Mass spectrometry

LC–MS/MS analysis was carried out using an Orbitrap Fusion™ mass spectrometer (Thermo Fisher Scientific) with a New Objective digital PicoView 565 nanospray source (Scientific Instrument Services, Palmer, MA, USA) coupled to a Dionex™ UltiMate™ 3000 RSLC Nanoliquid chromatograph. The peptides were loaded into an Acclaim PepMap™ 100 nano trap column (nanoViper™ C18, 0.3 × 5 mm, 5 μm particle size, 100 Å pore size; Thermo Fisher Scientific) with loading buffer (2% ACN with 0.05% aqueous TFA (v/v)) for 5 min desalting at a flow rate of 5 μL·min^−1^. Next, the peptides were eluted onto an Acclaim PepMap™ RSLC C18 (nanoViper™ 75 μm × 25 cm, 2 μm particle size, 100 Å pore size; Thermo Fisher Scientific) kept at 50 °C and separated by linear gradient elution over 36 min from 2% to 25% B and a 5‐min gradient from 25% to 60% B, followed by a 6‐min wash step with 98% B, and 27 min of equilibration with 2% B. Mobile phase A was composed of LC–MS grade water and 0.1% formic acid (FA), while B was 80% acetonitrile with 0.1% aqueous FA (v/v). The flow rate was 300 nL·min^−1^. The Orbitrap mass analyser was operated in positive ion mode, with the static positive ion spray voltage set to 2.7 kV and the ion transfer tube temperature set to 275 °C. The master scan was acquired at resolving power settings of 120 000 (FWHM at *m/z* 200); the precursor mass range was 350–1400 *m/z*. The MS/MS spectra of multiply charged ions were collected in data‐dependent mode (fragment and measure most intensive precursors for 2 s). Dynamic exclusion was set to 30 s. The peptides were fragmented using higher‐energy collisional dissociation (HCD) with the normalised collision energy setting at 30% and the isolation window of 4 Da. The peptide fragments generated via HCD were detected in an ion trap (rapid scan rate).

#### 2.9.3 Data processing

Data analysis was performed with peaks studio 11 (Bioinformatics Solutions Inc., Waterloo, ON, Canada). The raw data were treated with mass correction and chimera associations. The data were then searched by the Peaks search engine where the main database was *Homo sapiens* proteins from Swiss‐Prot with the addition of p21^L^ sequence; the secondary database was common MS contaminants. Precursors were searched with 10 p.p.m. mass tolerance and fragments with 0.6 Da mass tolerance. The enzyme parameter was set as semi‐specific trypsin with up to three missed cleavages. A total of four variable modifications were considered: acetylation on protein N terminus, carbamidomethylation on cysteine, deamidation of asparagine or glutamine, and methylation on methionine. Results were validated by a decoy search used to set the false discovery rate on the peptide level to 1%.

The mass spectrometry analysis was performed using a single biological replicate for each sample.

## 3 Results

### 3.1 CDK11 inhibition leads to rapid p53 stabilisation

**Fig. 1:**
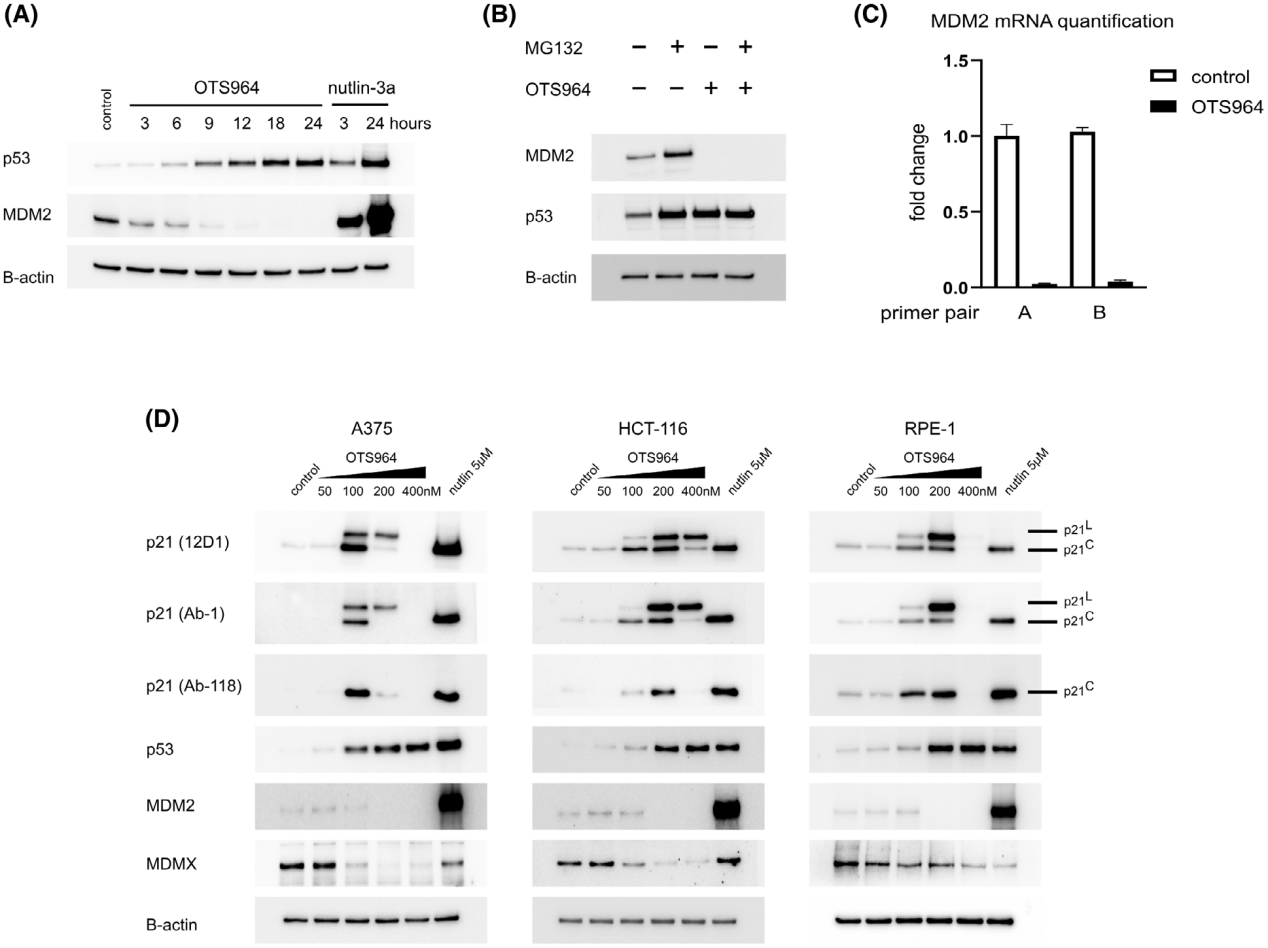
CDK11 inhibition stabilises p53 and induces canonical and alternative p21 isoforms. (A) p53 and MDM2 levels were assessed with immunoblotting in A375 cells treated with 200 nm OTS964 (3, 6, 9, 12, 18, 24 h) or 5 μm nutlin‐3a (3, 24 h). β‐actin served as a loading control. *n* = 3 biologically independent replicates. (B) The effect of MG132 proteasome inhibitor on MDM2 and p53 protein levels in A375 cells treated with OTS964 (200 nm) for 24 h. MG132 (20 μm) was added to the indicated samples for the last 3 h. β‐actin served as a loading control. *n* = 3 biologically independent replicates. (C) MDM2 mRNA levels in A375 cells treated with OTS964 (200 nm, 24 h) were assessed with RT‐qPCR using primers targeting different transcript regions. Primer pair A: nt 958–1069, primer pair B: nt 254–366 of the full‐length canonical MDM2 transcript (NCBI Reference Sequence: NM_002392.6). *n* = 3 biologically independent replicates, each with four technical replicates (mean and SD shown). (D) A375, HCT‐116, and RPE1 cells were treated with a range of OTS964 concentrations or nutlin‐3a (positive control) for 24 h. Three different monoclonal antibodies were used for the detection of p21 (12D1, Ab‐1, Ab‐118) with immunoblotting. β‐actin served as a loading control. 12D1 and Ab‐1 antibodies show two isoforms of p21, termed p21^C^ and p21^L^. In addition, p53, MDM2 and MDMX levels were assessed. *n* = 3 biologically independent replicates.

A375 melanoma cells with WT p53 were treated with OTS964 (200 nm) in a time‐course experiment, and the levels of p53 were assessed with immunoblotting. The results showed p53 induction 6 h after treatment, with a further increase later in time (Fig. 1A). The effect was slower than that of nutlin‐3a, used as a positive control for p53 stabilisation, which showed substantial induction as soon as 3 h after treatment. However, p53 levels were similar in samples treated with OTS964 or nutlin‐3a after 24 h. Subsequently, MDM2 levels were assessed, showing a decrease as soon as 3 h after OTS964 treatment and a complete loss of signal after 12 h (Fig. 1A). This contrasts with the effect of nutlin‐3a, which induces MDM2 in a negative feedback loop.

MDM2 has a half‐life as short as 5 min in stress conditions due to rapid ubiquitination and proteasomal degradation [46]. To test whether MDM2 downregulation is caused by accelerated degradation or suppressed synthesis, we assessed the effect of the proteasome inhibitor MG132 on MDM2 levels. In our experiment, cells were treated with OTS964 for 24 h and MG132 was added for the last 3 h. The results showed that MG132 could not stabilise MDM2 levels after CDK11 inhibition, indicating that MDM2 was not downregulated by proteasomal degradation (Fig. 1B). In the next step, we analysed the levels of transcription of the *MDM2* gene with RT‐qPCR using two primer pairs targeting different regions of the transcript. In both cases, the results showed substantially suppressed *MDM2* mRNA levels in OTS964 samples, which suggested downregulation of *MDM2* transcription or degradation of the mRNA (Fig. 1C).

### 3.2 CDK11 inhibition induces canonical and alternative p21 isoforms

Knowing that p53 is upregulated by CDK11 inhibition, we investigated if p53 activates its typical downstream target, p21. Using immunoblotting, we demonstrated induction of p21 in A375 cells and, interestingly, an additional band with lower electrophoretic mobility, which was not present in nutlin‐3a treated samples (Fig. 1D). To discover if the slower migrating band is specific for the A375 cell line, we tested two other cell lines, HCT‐116 (colorectal carcinoma, p53 WT) and RPE1 (noncancerous retinal epithelium). Three different monoclonal antibodies were used to test if the band was a variant of the p21 protein or a result of the cross‐binding of the antibody. Two antibodies, 12D1 and Ab‐1, showed the secondary band in all three cell lines in OTS964‐treated samples, while Ab‐118 showed only the typical 21 kDa band (Fig. 1D). The fact that two different monoclonal antibodies recognised the secondary band suggested that the signal was specific and that the extra band represents a p21 variant. At the same time, the lack of Ab‐118 binding to the slower migrating band indicated a missing or masked epitope in this variant. The levels of the typical and the slower migrating variants were OTS964 concentration‐dependent; lower concentrations predominantly induce the typical p21, moderate concentrations induce both variants, and higher concentrations predominantly induce the slower migrating variant. The exact OTS964 concentrations causing these effects differ between the cell lines. The highest concentration used in this study (400 nm) caused complete loss of both variants in A375 and RPE1 cell lines (not HCT‐116) despite high levels of p53 present in cells (Fig. 1D). p53 and MDM2 proteins were also assessed in HCT‐116 and RPE1 cell lines, and the results showed changes in protein levels similar to those observed in A375 cells. Additionally, analysis of MDMX, the second key p53 regulator, revealed downregulation after OTS964 treatment in all cell lines (Fig. 1D).

**Fig. 2:**
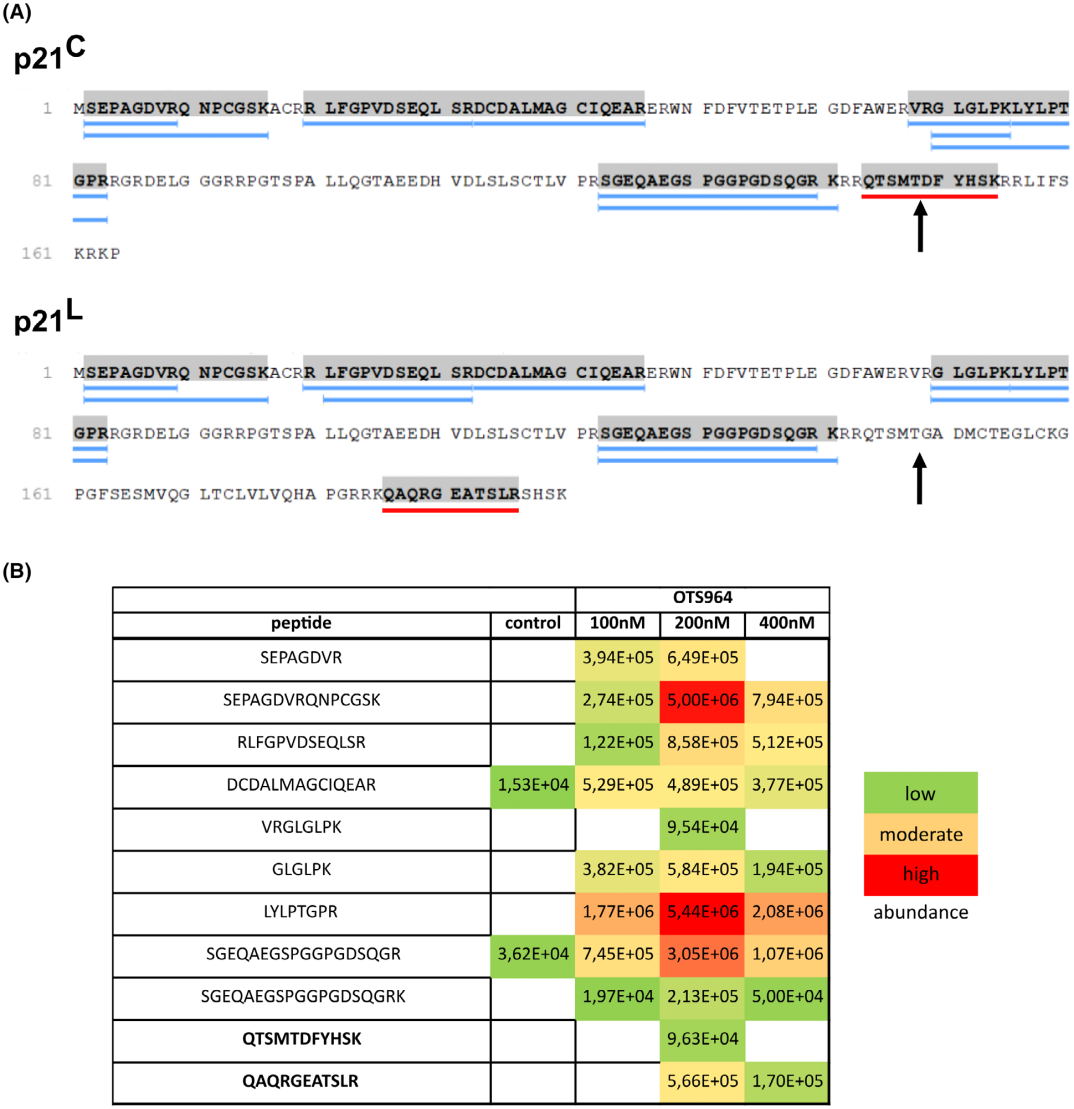
Mass spectrometry analysis of p21 isoforms. (A) Sequences of p21^C^ and p21^L^. Peptides that were identified in the mass spectrometry analysis are underlined, in blue if the peptide is common for both isoforms and in red if it is unique for either isoform. The sequences preceding the black arrows are identical in both isoforms, whereas the sequences following the arrows are isoform‐specific. (B) Relative quantification of the p21 peptides identified in HCT‐116 samples. HCT‐116 cells were either untreated or treated with 100, 200 or 400 nm OTS964 for 24 h. Shading indicates the abundance of the peptide in that particular sample relative to all other p21‐specific peptides identified in this analysis; an empty box indicates that the peptide was not identified.

After searching the literature, we found a possible candidate for the alternative p21 variant in the p21^L^ isoform [36]. p21^L^ lacks the C‐terminal 16 AA of canonical p21, hereinafter referred to as p21^C^, and instead has an extra 52 AA originating from what is considered an intron sequence usually removed on the spliceosome (Fig. 2A). To test whether our variant is indeed p21^L^, we applied mass spectrometry proteomic analysis. For this experiment, HCT‐116 cells were used because we had identified experimental conditions that induced predominantly p21^C^ after 24 h of OTS964 (100 nm), or predominantly p21^L^ (400 nm), with a mixture of the two variants at 200 nm in this cell line (Fig. 1D). We identified and quantified unique peptides that were only present in one of the two p21 isoforms and, at the same time, not present in any other protein in the human proteome according to the Swiss‐Prot database. The p21^L^‐specific peptide QAQRGEATSLR was found only in samples treated with 200 and 400 nm OTS964 (Fig. 2B), the same concentrations that showed strong p21^L^ signals in the immunoblotting experiment (Fig. 1D). p21^L^ was also present in 100 nm samples in immunoblotting, although it was weaker compared to 200 and 400 nm, and the abundance of p21^L^ might have been below the identification threshold in our proteomic analysis. Analogously, the p21^C^ peptide QTSMTDFYHSK was identified only in cells treated with 200 nm OTS964 (Fig. 2B), where p21^C^ levels were highest (Fig. 1D).

### 3.3 Epitope identification of p21 antibodies

**Fig. 3:**
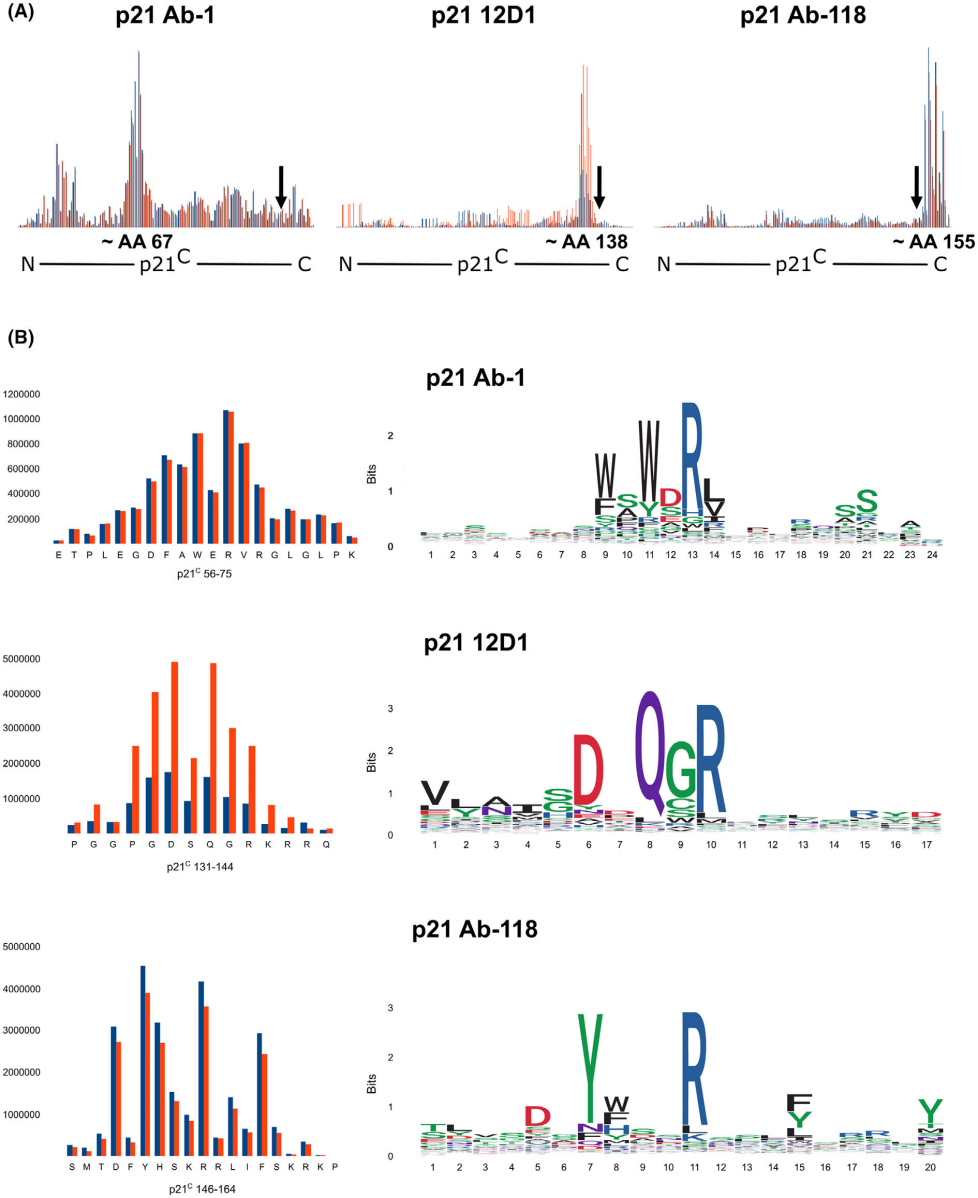
The epitopes of the p21 antibodies. Epitopes were mapped using phage display. (A) Number of sequenced reads (*y*‐axis) aligned to the sequence of p21^C^ (*x*‐axis). Regions with the highest number are the most likely to form the epitope. Results from two technical replicates are displayed separately in red and blue. The black arrows indicate the position of the last common AA of p21^C^ and p21^L^. (B) On the left, regions with the highest number of reads are displayed. On the right are the sequence logos generated by template‐free, independent‐clustering‐based analysis, showing a virtual sequence created by aligning the sequencing reads. Multiple AAs are shown in each position, the size of the letter reflects the abundance of that particular AA in that position.

**Fig. 4:**
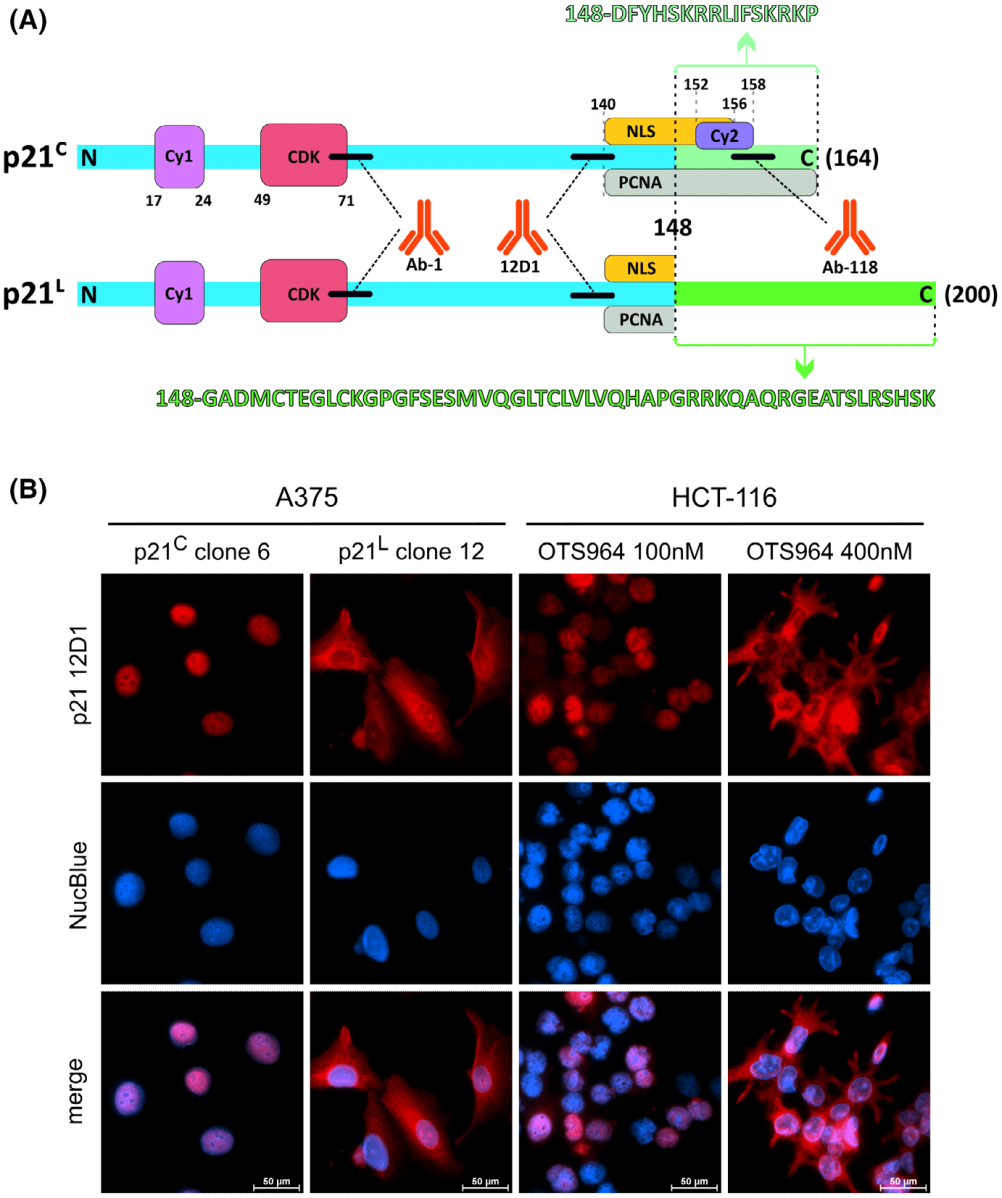
Structural and functional differences in p21 isoforms. (A) The diagram shows p21^C^ (164 AA) and p21^L^ (200 AA) isoforms. The common part of the proteins (148 AA) is displayed in blue, while the alternative C terminus is green. The functional motifs discovered in p21^C^ are shown and numbers indicate their position in the protein sequences; CDK‐binding motif, cyclin‐binding Cy1 and Cy2, PCNA‐binding PIP box motif and NLS. The black lines indicate the position of the epitopes of the antibodies used in this study. While the epitopes of Ab‐1 and 12D1 are present in both isoforms, the C‐terminal Ab‐118 epitope is missing in p21^L^. (B) Immunofluorescence images of p21 isoforms in cells. A375 cells (left panel) stably transfected with doxycycline‐inducible p21^C^ (clone 6) and p21^L^ (clone 12) constructs were labelled with anti‐p21 12D1 and fluorescent secondary antibody. p21 is shown in red, and the cell nuclei labelled with NucBlue in blue. As evident from merged images, individual isoforms occupy different locations in cells, with p21^C^ colocalising with nuclei and p21^L^ showing cytoplasmic localisation. In addition, subcellular localisation of p21 isoforms was assessed in HCT‐116 cells (right panel) in which p21 expression was induced by OTS964 (100 nm concentration for p21^C^ and 400 nm concentration for p21^L^ as determined in Fig. 1D). The distribution of both isoforms is similar to that in A375 cells with doxycycline‐inducible p21. Scale bar = 50 μm, *n* = 3 biologically independent replicates.

The determination of antibody epitopes is pivotal for studies of protein isoforms. Therefore, we identified the epitopes of the p21 antibodies used in this study using the phage display method. With this approach, we discovered that the two antibodies that bind to both p21 isoforms in immunoblotting (12D1, Ab‐1) recognise epitopes localised within the common part of p21^C^ and p21^L^. The epitope of the p21^C^‐specific antibody Ab‐118 lies in the variable C‐terminus of the p21^C^ isoform (Fig. 3). The position of the epitopes and functional motifs in the p21 isoforms is illustrated by the diagram in Fig. 4A.

### 3.4 Subcellular localisation of p21 isoforms

In the previous study [36], the authors showed that p21^L^ does not accumulate in the nucleus, which is the typical location for p21^C^. To test this, clones of A375 cells were prepared with doxycycline‐inducible p21^C^ or p21^L^ incorporated in their genome, and cells were labelled with anti‐p21 12D1 antibody for immunofluorescence microscopy after induction. Our results support earlier observations, as cells expressing p21^C^ showed nuclear signal, while cells expressing p21^L^ showed predominantly cytoplasmic signal (Fig. 4B). To further assess whether the isoforms exhibit the same subcellular localisation in OTS964‐treated samples, HCT‐116 cells were labelled with anti‐p21 12D1 after induction of p21 isoforms with different OTS964 concentrations, as determined in Fig. 1D. Cells treated with 100 nm concentration, inducing high p21^C^/low p21^L^ expression, showed predominantly nuclear signal, while cells treated with 400 nm OTS964, inducing low p21^C^/high p21^L^ expression, showed predominantly cytoplasmic signal (Fig. 4B).

### 3.5 p21^L^ is produced as a result of SF3B1 inhibition in the presence of p53

**Fig. 5:**
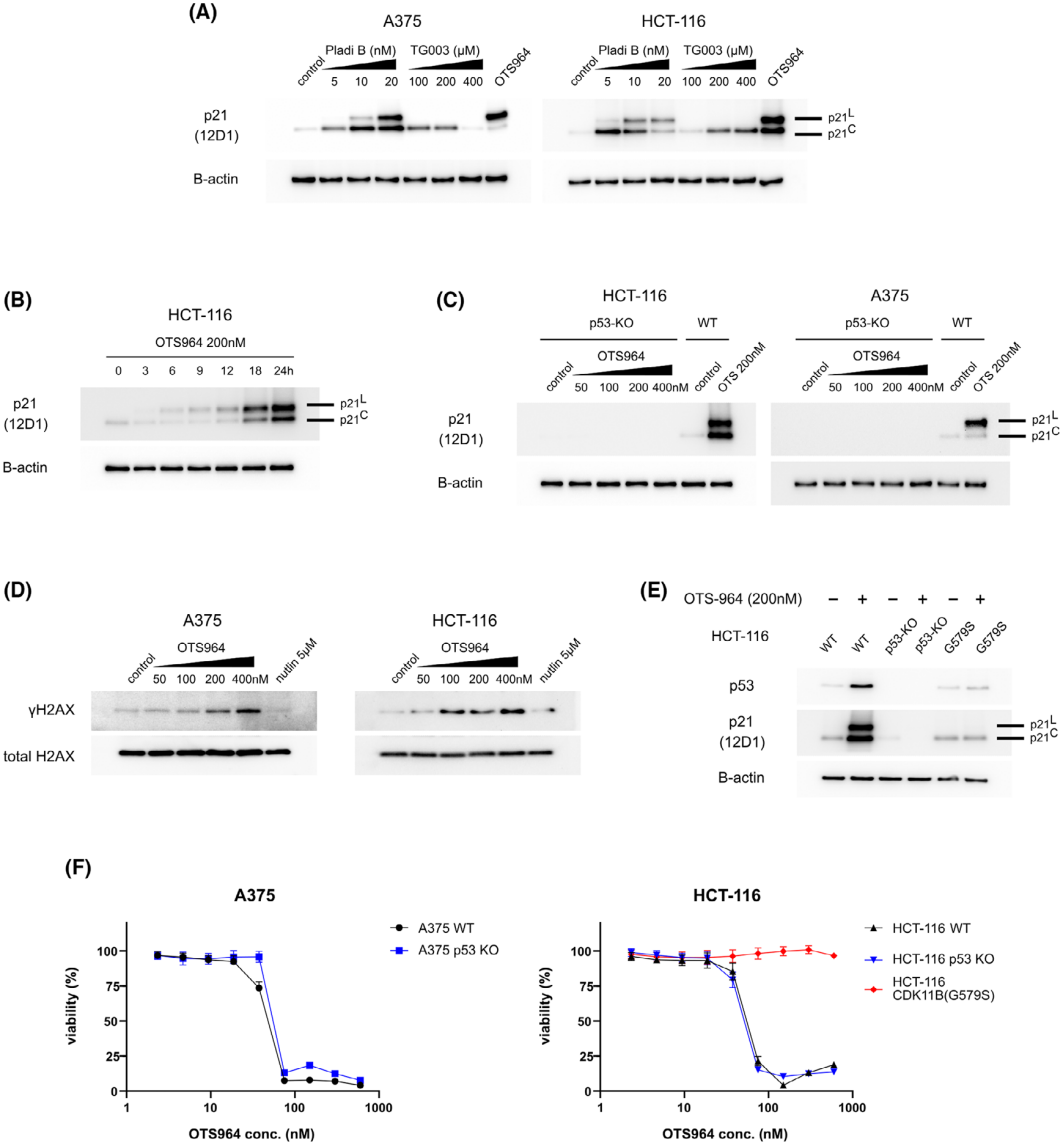
CDK11 inhibition induces p21^L^ by p53 stabilisation and SF3B1 inactivation. (A) A375 and HCT‐116 cells were treated with increasing concentrations of pladienolide B (Pladi B) and TG003 for 24 h and the levels of p21^C^ and p21^L^ were assessed with immunoblotting using 12D1 antibody; β‐actin served as a loading control. OTS964 (200 nm) was used as a positive control. *n* = 3 biologically independent replicates. (B) HCT‐116 cells were treated with 200 nm OTS964 and harvested at different time points. The levels of p21 isoforms were assessed using 12D1 antibody along with β‐actin (loading control). *n* = 3 biologically independent replicates. (C) A375 p53‐KO and HCT‐116 p53‐KO cells were treated with a range of OTS964 concentrations for 24 h and the protein levels of p21^C^ and p21^L^ were assessed using 12D1 antibody; β‐actin served as a loading control. HCT‐116 WT and A375 WT cell lines were used as positive controls. *n* = 3 biologically independent replicates. (D) γH2AX and total H2AX levels in cells treated with OTS964. A375 and HCT‐116 cells were treated with a range of OTS964 concentrations and nutlin‐3a for 24 h. *n* = 3 biologically independent replicates. (E) The effect of CDK11B(G579S) substitution on the p53 and p21 induction in cells treated with OTS964. HCT‐116, HCT‐116/CDK11B(G579S) and HCT‐116 p53‐KO cells were treated with OTS964 (200 nm) for 24 h and their levels of the p53 protein and p21 isoforms were assessed. *n* = 3 biologically independent replicates. (F) The effect of p53 on cell viability upon OTS964 treatment. WT and p53‐KO A375 and HCT‐116 cell lines were treated with OTS964 (4–600 nm) for 72 h and cell viability was determined with the resazurin assay. The difference between WT and p53‐KO variants represents the p53 contribution to OTS964‐induced toxicity for each cell line. HCT‐116/CDK11B(G579S) was also analysed to show the effect of CDK11B(G579S) substitution on sensitivity to OTS964. *n* = 3 biologically independent replicates, each with six technical replicates (mean and SD shown).

It has been shown that p21^L^ is produced in cells treated with E7107, an inhibitor of SF3B1 [36]. Based on our results, we assumed that OTS964 similarly induces p21^L^ by blocking SF3B1 functions indirectly by preventing CDK11‐mediated SF3B1 phosphorylation [17]. Therefore, we aimed to determine if p21^L^ induction is specific for SF3B1 inhibition or can be elicited by targeting different spliceosome components. TG003 is a splicing modulator that inhibits Cdc2‐like kinases (CLK) and prevents phosphorylation of the SF2/ASF splicing factor [47]. This compound induced p21^C^ in A375 and HCT‐116 cell lines (Fig. 5A), which is expected because disruption of splicing is known to activate the p53 response [48]. However, TG003 did not induce p21^L^, indicating that this is not a regular consequence of defective spliceosome functioning and that not all insults to the splicing machinery lead to the alternative splicing of *CDKN1A* mRNA to produce p21^L^. In contrast, pladienolide B, a second SF3B1 inhibitor similar to E7107, induced both p21 isoforms in A375 and HCT‐116 cells (Fig. 5A).

A time‐course experiment was performed to investigate the dynamics of p21^C^/p21^L^ accumulation. HCT‐116 cells were treated with 200 nm OTS964, which causes the accumulation of comparable amounts of both isoforms after 24 h (Fig. 1D), and harvested at six different time points (3–24 h). p21^C^ levels were lower than control for the first 12 h of incubation, while p21^L^ gradually accumulated. At later time points, a substantial amount of p21^C^ accumulated along with more p21^L^ (Fig. 5B). This result suggests that the hypothesis of gradual spliceosome incapacitation by OTS964, culminating with complete loss of function and subsequent defective splicing of *CDKN1A* mRNA, may not fully explain our observations. Instead, the data indicate that 200 nm OTS964 appears to disrupt the splicing machinery sufficiently to allow simultaneous synthesis of both isoforms in cells, though further investigation would be needed to confirm this mechanism.

Because p21 transcription is induced in response to p53 activation and p53 is activated by OTS964, we assumed that p53 knockout cells would not show induction of either p21 isoform after treatment with OTS964. Therefore, we employed A375 and HCT‐116 *TP53*‐knockout cells (A375 p53‐KO, HCT‐116 p53‐KO) treated with OTS964. There was no induction of either p21 isoform, confirming that p21^L^ induction, like p21^C^ induction, is p53‐dependent (Fig. 5C).

### 3.6 γH2AX induction indicates DNA damage after CDK11 inhibition

Because the main function of p53 is to maintain genome integrity and DNA damage is a major stimulus for p53 activation, we assessed levels of DNA double‐strand break marker γH2AX after CDK11 inhibition [49]. Increased γH2AX was detected in both A375 and HCT‐116 cell lines treated for 24 h with OTS964 at concentrations as low as 50 nm (Fig. 5D). This finding suggests that, in addition to disrupting RNA splicing or, as a consequence of that, CDK11 inhibition is harmful to the genome, p53 is likely activated due to the occurrence of DNA double‐strand breaks.

### 3.7 OTS964‐resistant CDK11B(G579S) prevents p53 activation

Specific inhibition of a single CDK with a low‐molecular‐weight inhibitor is often problematic, and most inhibitors show activity against multiple CDKs. Even though OTS964 was shown to be exceptionally selective [16, 17], we investigated if the effects of OTS964 on p53 are solely caused by CDK11 inhibition. For these experiments, we used homology‐directed repair (HDR)‐mediated gene editing to prepare HCT‐116 cells harbouring the G579S substitution in the *CDK11B* gene, which prevents CDK11 inhibition with OTS964 [16]. The resulting HCT‐116/*CDK11B*(G579S) cell line is resistant to high doses of OTS964 and does not show induction of p53 or either p21 isoform (Fig. 5E,F). This indicates that p53 activation after OTS964 treatment is due to CDK11 inhibition and not by an undescribed side effect or by inhibition of another CDK.

### 3.8 Cytostatic/cytotoxic effects of CDK11 inhibition are p53‐independent

Considering that OTS964 elevates p53 levels and p53 induces cell cycle arrest and apoptosis, we assumed that p53 activation was at least partially responsible for the suppression of cancer cell viability treated with OTS964. To evaluate the contribution of p53, we compared the response to OTS964 of WT and p53‐KO A375 and HCT‐116 cells. The sensitivity to OTS964 was determined with the resazurin viability assay that captures the cytostatic and cytotoxic effects of a tested compound. Cells were treated with a range of OTS964 concentrations (4–600 nm), and their viability was assessed 72 h later. Unexpectedly, the results showed only a minor increase in resistance in A375 p53‐KO cells (IC50_WT_ = 49 nm vs. IC50_p53‐KO_ 68 nm) and no increase in HCT‐116 p53‐KO cells (IC50_WT_ = 54 nm vs. IC50_p53‐KO_ 49 nm) (Fig. 5F). Hence, despite rapid and substantial p53 induction, the effect of OTS964 is not primarily p53‐dependent, and other mechanisms are responsible for suppressed proliferation and/or cell death induced by CDK11 inhibition. Moreover, p53 activation appears to require OTS964 concentrations higher than the IC50 values observed in our viability assays.

### 3.9 p21^L^ isoform exerts an antiproliferative effect

**Fig. 6:**
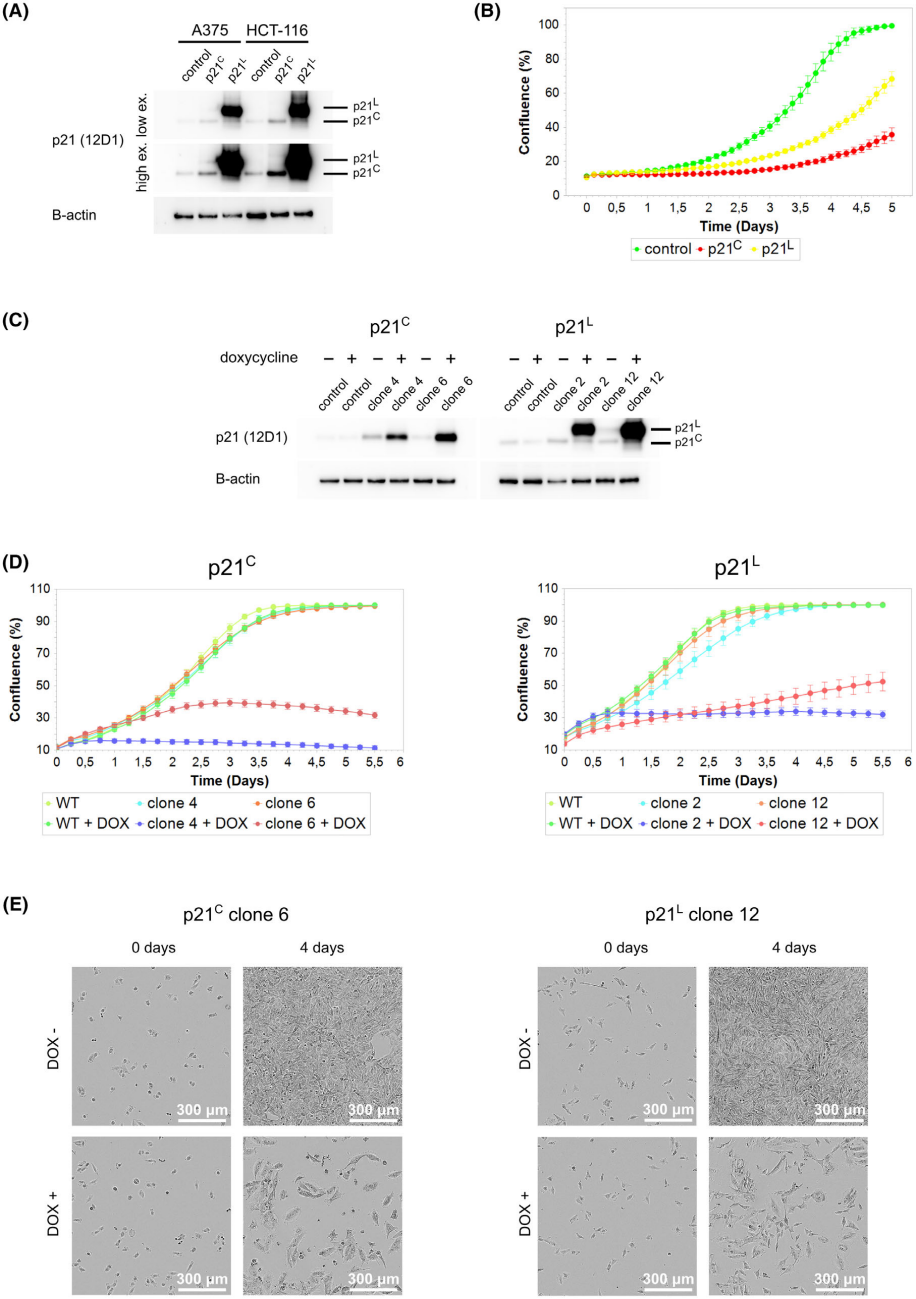
Both p21 isoforms inhibit cell proliferation. (A) Immunoblotting analysis of p21^C^ and p21^L^ levels in A375 and HCT‐116 cells after the transfection with p21^C^ and p21^L^ expression vectors. The high and the low exposure images of the p21 membranes are shown. *n* = 3 biologically independent replicates. (B) A375 cells were transfected with p21^C^ or p21^L^ (GFP transcriptional fusion) or GFP expression vectors (control), the cells with high GFP signal were sorted with FACS on 96‐well plates and their confluence was measured every 3 h for 5 days with an automated microscopy system. The graph shows the changes in cell confluence over time. *n* = 3 biologically independent replicates, each with two technical replicates (mean and SD shown). (C) Confirmation of efficacy of p21^C^ and p21^L^ expression in A375 cells stably transfected with doxycycline‐inducible p21^C^ and p21^L^ constructs. Two cell clones were tested for each isoform along with WT A375 cells as a control. The cells were either treated or not with 5 μg·mL^−1^ doxycycline. *n* = 3 biologically independent replicates. (D) A375 cells with doxycycline‐inducible expression of p21^C^ and p21^L^ were seeded on 96‐well plates with or without the addition of doxycycline to the culture medium and for 5 days, their confluence was measured every 6 h with an automated microscopy system. Two clones were tested for each isoform along with WT A375 cells which served as a control. *n* = 3 biologically independent replicates, each with 6 technical replicates (mean and SD shown). (E) Microscopic images of cells from D, p21^C^ clone 6 and p21^L^ clone 12, taken at the time of induction with doxycycline (0 days) and 4 days later (4 days). Scale bar = 300 μm.

The biological properties of the p21^L^ isoform could help to explain why p53 does not substantially affect cell viability upon CDK11 inhibition. To replicate earlier observations of p21^L^ properties [36], we transfected A375 cells with p21^C^ and p21^L^ cDNAs and measured the effects on cell proliferation. Transcriptional fusion of p21 and GFP allowed us to select the cells with high p21 expression based on high GFP fluorescence signal without compromising p21 structure by direct tagging. The cells were sorted using fluorescence‐activated cell sorting (FACS) 24 h after transfection. The efficiency of the transfection was confirmed by immunoblotting (Fig. 6A). Interestingly, p21^L^ showed higher protein levels than p21^C^, even though the cDNAs were under the control of the same EF‐1α promoter in the same plasmid. An empty vector with GFP only was used as a control. The cells were monitored using an automated microscopy system (Incucyte S3; Sartorius AG) every 3 h for 5 days. Contrary to expectations, both isoforms suppressed proliferation, although the effect of p21^L^ was significantly reduced (Fig. 6B).

Because our results did not agree with previous data that p21^L^ does not cause cell cycle arrest [36], we continued testing with a second experimental method, the same that was used in the previous study. Clones of A375 cells were used with doxycycline‐inducible p21^C^ or p21^L^, and proliferation was measured in the presence or absence of doxycycline. The efficiency of the p21 induction was confirmed by immunoblotting, which also confirmed our observations that p21^L^ accumulates to higher levels in cells (Fig. 6C). The proliferation measurements with the Incucyte showed inhibition of proliferation by both isoforms (Fig. 6D). Although comparing the effect of the two isoforms would require testing a high number of clones to be statistically relevant, the inhibitory effect of both isoforms is obvious from our data. The microscopic analysis also showed swelling of cells after induction of both p21 isoforms, which was likely responsible for any increase in confluence at later time points (Fig. 6E).

Based on our findings, we conclude that p21^L^ induces cell cycle arrest, although its inhibitory capacity is lower than that of p21^C^. A possible explanation is that p21^L^ lacks the PIP box and the Cy2 motifs important for blocking the cell cycle, but the Cy1 and CDK‐binding motifs are preserved to provide partial inhibitory activity. Higher protein levels of p21^L^ than p21^C^ that we observed in cells might have been an important factor partially compensating for the loss of p21^L^ activity in our experiments.

### 3.10 OTS964 induces p21^L^ in murine cells

**Fig. 7:**
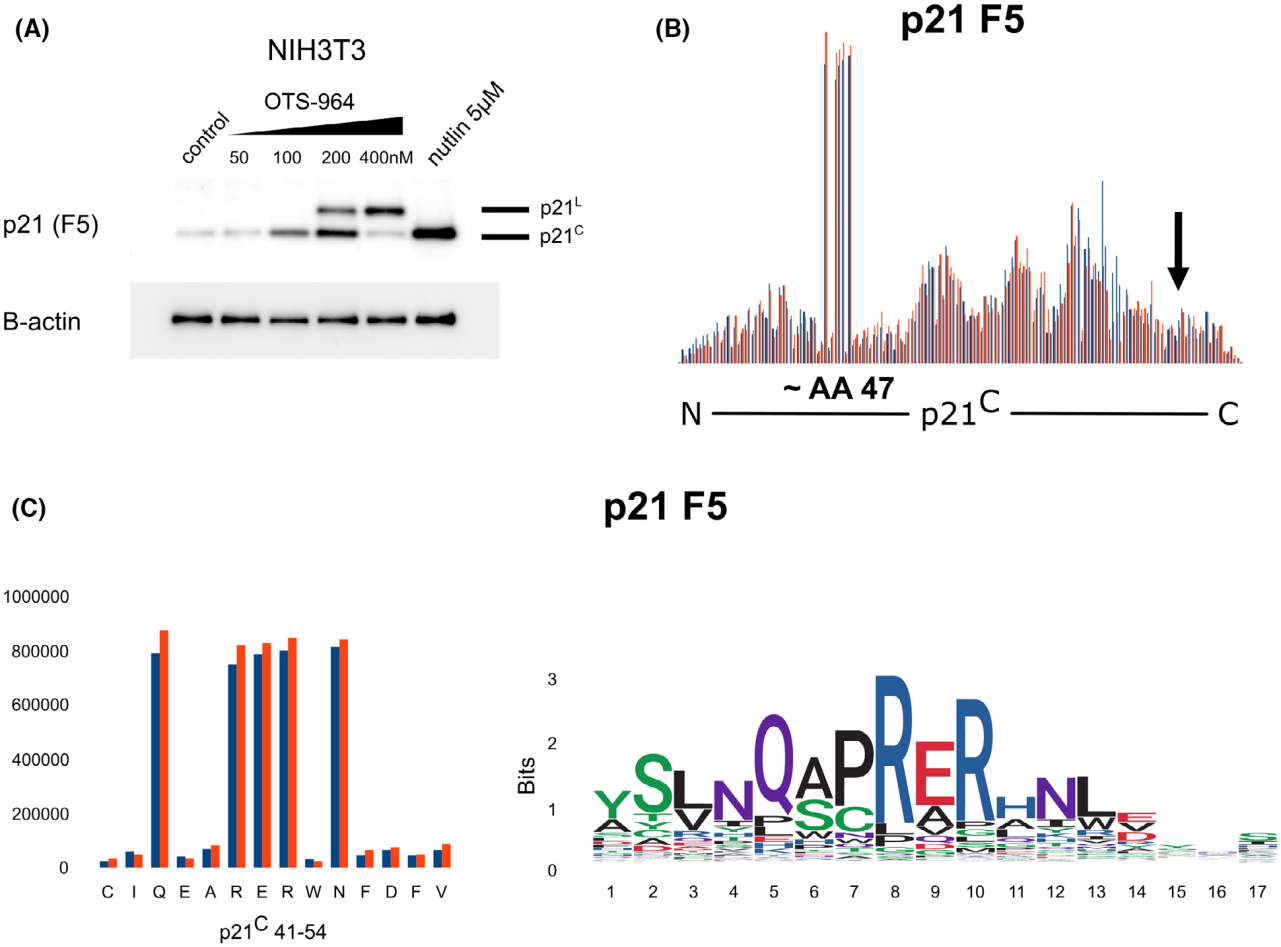
Detection of p21 isoforms in murine fibroblast cells treated with OTS964. (A) Murine NIH3T3 cells were treated with a range of OTS964 concentrations and nutlin‐3a, which served as a positive control for the induction of the canonical murine p21 protein (p21^C^). After 24 h of incubation, p21 was detected with immunoblotting using the F5 antibody along with β‐actin (loading control). *n* = 3 biologically independent replicates. (B) Epitope identification of the F5 antibody. The epitope was mapped using phage display; a graph from software‐aligned sequencing reads to p21^C^ displays the number of reads on the *y*‐axis aligned to each position on the *x*‐axis. The region with the highest number of reads is most likely to form the epitope. Results from two technical replicates are displayed separately in red and blue. The black arrows indicate the position of the last common AA of p21^C^ and p21^L^. (C) On the left, the region with the highest number of reads is shown in more detail. On the right are the sequence logos generated by template‐free, independent‐clustering‐based analysis, showing a virtual sequence created by aligning the sequencing reads. Multiple AAs are shown in each position, the size of the letter reflects the abundance of that particular AA in that position.

The level of evolutionary conservation reflects the importance of a given mechanism or molecule. Therefore, we investigated if an isoform similar to p21^L^ is produced in cells from a different species. For this experiment, murine cells were selected because of the suitable evolutionary distance between the species, the availability of an antibody, and because mouse is the most widely used model organism in biomedical research, with the potential for producing transgenic models in the future. The murine homologue of the *CDKN1A* gene, *Cdkn1a*, has a similar architecture to the human gene. It has two protein‐coding exons and, if the intron between them is not spliced out, the transcript continues in‐frame potentially giving rise to a longer isoform with an alternative C terminus. NIH3T3 murine fibroblasts were treated with OTS964 and p21 was detected with immunoblotting, showing two bands with sizes that matched canonical murine p21 and the theoretical product analogous to human p21^L^ predicted by our analysis of the *Cdkn1a* gene, which was seen only in OTS964 treated cells (Fig. 7A). An analysis of the epitope of the F5 antibody used in this experiment revealed that the antibody recognises the N‐terminal epitope present in the canonical isoform and the predicted alternative isoform (Fig. 7B,C).

## 4 Discussion

We discovered that pharmacological inhibition of CDK11 with OTS964 stabilises p53 and induces p53‐dependent production of an alternative p21^L^ isoform, and that p53 stabilisation is caused by MDM2 downregulation either by *MDM2* promoter silencing or mRNA degradation. Elevated levels of γH2AX, indicative of DNA damage, were detected, suggesting that CDK11 inhibition causes genomic lesions, representing the primary stimulus for p53 stabilisation. Alternatively, defective splicing of *MDM2* mRNA could play a role, as was observed earlier after SF3B1 inhibition by the E7107 compound [36]. However, a comparison of the effects of OTS964 on cells with and without functional *TP53* demonstrates that the substantial impact of OTS964 on cancer cell viability is not related to its ability to activate the p53 response.

We focused on p21^L^ more deeply because it was described only recently and is possibly the only alternative p21 isoform documented on the protein level yet. p21^L^ was initially discovered in cells treated with the E7107 inhibitor of SF3B1, which is a crucial spliceosomal protein that needs to be phosphorylated by CDK11 to execute its functions in RNA splicing [36]. Consistent with that, we showed that p21^L^ is induced by blocking SF3B1 by the upstream‐acting compound OTS964 that inhibits CDK11. Moreover, we showed that p21^L^ induction is not a common response to spliceosome disruption, as the TG003 splicing inhibitor targeting CLK induces only p21^C^. Our results agree with earlier observations that spliceosome disruption activates p53 and induces p21^C^ [48], although here we report p53‐dependent induction of p21^L^ that is specific for inhibition of SF3B1 functions.

In a previous study, the authors showed that, unlike p21^C^, p21^L^ is preferentially located in the cytoplasm due to an incomplete NLS, and that it lacks the ability to elicit cell cycle arrest [36]. However, our data only support the former finding regarding subcellular localisation. Using two experimental approaches, we determined that p21^L^ suppresses proliferation in A375 cells, even though its inhibitory capacity is lower than that of p21^C^. Among the possible reasons for the diminished activity of p21^L^ are the absence of functional motifs responsible for binding PCNA and cyclins (PIP box and Cy2), and cytoplasmic localisation, since nuclear interaction partners are unavailable for p21^L^.

Although p21^L^ is not inactive, the switch from p21^C^ to p21^L^ could provide a growth advantage for cancer cells due to its partial loss of function, and p21^L^ may be responsible for some cases of unusual behaviour of p21. p21^L^ is hard to distinguish from p21^C^ in immunoblotting when an antibody is used that recognises both isoforms (12D1, Ab‐1) and is indistinguishable in cell/tissue staining. If a C‐terminal p21 antibody is used (Ab‐118), p21^L^ is not detectable. While p21^C^ is predominantly a nuclear protein, it is not rare for p21 to be observed in the cytoplasm, the expected location of p21^L^, and this location has clinical relevance in ovarian and breast cancer [50, 51]. Cytoplasmic accumulation of p21 is generally attributed to its phosphorylation on T145 and other residues [28, 34]. However, p21^L^ might be responsible for some of the observations where p21 phosphorylation status was not assessed. The ability of p21^L^ to be phosphorylated on T145 is yet to be determined. We assume that mutations of SF3B1 could lead to p21^L^, and therefore, p21^L^ could be present in tumours with these mutations.

An important question is how is p21^L^ synthesised and what the exact RNA template is? Simple intron retention would result in the mRNA having a premature termination codon that might be a target of nonsense‐mediated decay. If p21^L^ is a product of natural alternative splicing, then its mRNA undergoes a regulated splicing event and is compliant with all the requirements of mRNA quality control mechanisms, except that the splicing takes place at an alternative splice site compared to p21^C^. An alternative explanation is that shorter pre‐mRNA molecules can escape mRNA surveillance mechanisms, translocate to the cytoplasm, and be translated [52, 53]. This might apply to p21, as *CDKN1A* is a relatively small gene with three exons coding for a 164 (or 200) AA‐long protein. Interestingly, SF3B1‐targeting inhibitors pladienolide B and spliceostatin A cause intron retention in *CDKN1B* mRNA, which codes for p27, a related member of the CIP/KIP family, resulting in the p27* protein with an alternative C terminus and increased stability [53, 54]. This supports the idea of a mechanism that senses RNA splicing disruption and orchestrates the cellular response, involving proteins from the CIP/KIP family [54].

We observed a protein similar to p21^L^ in murine cells treated with OTS964, which indicates some level of evolutionary conservation and raises the question as to whether p21^L^ is produced in healthy cells under specific circumstances. The presence of p21^L^‐like proteins in multiple species suggests that p21^L^ is not only induced by artificial splicing dysregulation but may serve a function in normal cell physiology. However, the precise contexts in which p21^L^ might be produced and its significance remain to be determined. Furthermore, future work will elucidate the role of p21^L^ in cancer and whether this isoform has clinical relevance. Our identification of epitopes of widely used p21 antibodies and discovery of their binding abilities for p21 isoforms provide valuable information that will facilitate further investigations.

## 5 Conclusions

Our findings demonstrate that CDK11 inhibition via OTS964 induces p53‐dependent production of the alternative p21^L^ isoform. Unlike previous reports, we show that p21^L^ possesses cell cycle‐inhibitory activity, albeit reduced compared to p21^C^. Moreover, the induction of p21^L^ represents a specific response to SF3B1‐related spliceosome disruption rather than a general splicing inhibition effect. The evolutionary conservation of p21^L^‐like proteins across species suggests potential physiological roles beyond pathological contexts. These results provide new insights into p21 biology and establish a foundation for future investigations into the clinical relevance of p21^L^ in cancer, particularly in tumours with SF3B1 mutations.

## Conflict of interest

The authors declare no conflict of interest.

## Author contributions

RK was responsible for the design of the study and writing the manuscript. He also contributed by preparing the plasmid vectors for transfections, performing viability assays, preparing OTS964‐resistant cells, and testing the inhibitory activity of p21 isoforms using Incucyte. LA performed immunoblotting experiments, prepared cells with inducible p21 expression, and operated the cell sorter. LM conducted RT‐qPCR experiments, analysed the effects of a proteasome inhibitor on MDM2 stability, and helped with interpreting results. VH performed antibody epitope mapping with phage display up to the point of sequencing phage DNA; OB was responsible for sequencing, and FZK analysed sequencing data and generated a graphical representation of the phage display data. OB prepared the diagram with the p21 isoforms. TH and LH were responsible for mass spectrometry sample preparation, measurements and data analysis. MK conducted the initial immunoblotting analysis of the effects of CDK11 inhibition on the p53 pathway. PZ analysed the subcellular localisation of p21 isoforms. PJC helped with the manuscript preparation. BV and DPL contributed to the design of the study and helped with interpreting results and manuscript revision.

## Data Availability

The mass spectrometry data have been deposited to the Proteome Xchange (PX) Consortium [55] via the Proteomics Identifications (PRIDE) partner repository [56] with the dataset identifier PXD057524.
